# Rolle der Social Media in der Radiologie

**DOI:** 10.1007/s00117-025-01444-y

**Published:** 2025-04-07

**Authors:** Lukas Müller, Mirjam Gerwing, Daniel Pinto dos Santos

**Affiliations:** 1 Klinik und Poliklinik für Diagnostische und Interventionelle Radiologie, Unimedizin Mainz, Langenbeckstr. 1, 55131 Mainz, Deutschland; 2 Universitätsinstitut für Diagnostische und Interventionelle Radiologie, Universitätsklinik OWL Bielefeld – Campus Klinikum Lippe, Detmold, Deutschland

Die Entwicklung sozialer Medien („Social Media“) ist nahezu so alt wie das Internet selbst. Mit der Verbreitung von Smartphones stiegen Nutzerzahlen und Interaktionen rasant an. Laut aktuellen Umfragen nutzen bereits mehr als 80 % der Arbeitnehmer:innen im Feld Radiologie soziale Medien an mehr als fünf Tagen die Woche [1]. Theoretisch ermöglichen soziale Medien als interaktive digitale Kanäle den Austausch vielfältiger Inhalte in unterschiedlichsten Formaten.

## Entwicklung und Wandel sozialer Medien

Soziale Medien haben sich in den letzten zwei Jahrzehnten von privaten Netzwerken zu vielseitigen Plattformen entwickelt. Während frühe Netzwerke wie MySpace (gegründet 2003) und Facebook (gegründet 2004) primär dem persönlichen Austausch dienten, etablierten sich LinkedIn & Co. zunehmend für berufliche Vernetzung und Karrierechancen.

Auch wissenschaftliche und medizinische Fachkreise nutzen Social Media gezielt für den Austausch. Neben textbasierten Plattformen wie X (ehemals Twitter) gewinnen audiovisuelle Formate (z. B. YouTube, Podcasts) an Bedeutung für Weiterbildung, interdisziplinäre Vernetzung und Karriereentwicklung.

In Tab. 1 wird eine Übersicht der relevantesten Social-Media-Plattformen und deren Hauptnutzen in der Radiologie, Vorteile der jeweiligen Plattform und mögliche Einschränkungen gegeben.

**Tab. 1:** Übersicht der relevantesten Social-Media-Plattformen und deren Hauptnutzen in der Radiologie, Vorteile der jeweiligen Plattform und mögliche Einschränkungen

Plattform	Hauptnutzen Radiologie	Vorteile	Einschränkungen
X (vormals Twitter)	Schnelle und einfache Kommunikation im Kurzformat	Echtzeit-Interaktion, Nutzung von Hashtags, Threads ermöglichen strukturierte Diskussionen	Zeichenbeschränkung, schneller Inhaltsumschlag, algorithmusabhängige Sichtbarkeit, Zunahme von Hassrede und Desinformation
Bluesky	Schnelle und einfache Kommunikation im Kurzformat	Offene, dezentrale Server mit zentraler Moderation für eine stärkere Kontrolle der Inhalte und des Umgangs zwischen den Nutzer:innen, weniger algorithmusabhängig, Alternative zu X	Noch geringe Nutzerbasis, eingeschränkte Funktionen im Vergleich zu X
Instagram	Teilen radiologischer Bilder, Falldiskussionen	Bild- und Kurzvideo-zentriert, beliebt bei jüngeren Nutzern, ermöglicht auch längere Videos durch Instagram-TV	Begrenzte Textkapazität, Datenschutzbedenken, algorithmusabhängige Sichtbarkeit
LinkedIn	Professionelles Networking, Karriereentwicklung	Beruflicher Fokus, Teilen von Inhalten, Publikation wissenschaftlicher Erfolge, zunehmende Sichtbarkeit in der radiologischen Gemeinschaft in den letzten Jahren	Begrenztes Engagement in einigen radiologischen Kontexten, algorithmusabhängige Sichtbarkeit
Facebook	Informelle Kommunikation, Gruppendiskussionen	Große Nutzerbasis, Gruppen für Fachkommunikation, verschiedene Inhaltsformate	Datenschutzbedenken, algorithmusabhängige Sichtbarkeit
YouTube	Lehrvideos, Tutorials, wissenschaftliche Präsentationen	Lange Videoinhalte möglich, breite Reichweite, nützlich für didaktische Inhalte	Zeitaufwendig in der Produktion, Sichtbarkeit abhängig von Algorithmen
TikTok	Kurzformatige Lehrinhalte	Interaktives, ansprechendes Format, erreicht jüngere Zielgruppen	Begrenzte professionelle Nutzung, eingeschränkte Inhaltstiefe, stark algorithmusabhängige Sichtbarkeit
Radiopaedia	Teilen und Bearbeiten von Lehrartikeln, radiologische Bilddatenbank	Hochwertige radiologische Inhalte, breite Community	Begrenzte allgemeine Reichweite, auf rein fachlichen Austausch ausgelegt
ResearchGate	Teilen wissenschaftlicher Arbeiten, Networking, Impact-Messung	Forschungsfokus mit breiter Sichtbarkeit	Begrenzte Nutzung außerhalb der akademischen Gemeinschaft
Mastodon	Fachbezogene Diskussionen in geschlossenen oder offenen Gruppen	Dezentral, werbefrei, keine algorithmische Bevorzugung von Inhalten, fachspezifische Instanzen möglich	Noch begrenzte Nutzerbasis, weniger Reichweite, teils fragmentierte Community
Threads	Austausch wissenschaftlicher Inhalte im Kurzformat	Stärkere Verbindung zu Instagram mit gut möglicher Kombination, einfach bedienbar, kein Zeichenlimit für längere Diskussionen	Noch geringe Verbreitung in der Wissenschafts-Community, algorithmusabhängige Sichtbarkeit

## Warum lohnt sich Social Media im professionellen Kontext?

Die schiere Menge an wissenschaftlichen Publikationen und Fachinformationen wächst rasant. In der heutigen „Attention Economy“ konkurrieren unzählige Inhalte um unsere begrenzte Aufmerksamkeit. Hier kann Social Media gezielt genutzt werden: Durch intelligente Algorithmen lassen sich relevante wissenschaftliche Arbeiten und Diskussionen teilen, sodass Fachleute gezielt auf für sie interessante Inhalte zugreifen können. Der Beitritt zu einer thematisch passenden „Bubble“ erleichtert den Zugang zu relevanten Studien, Expertenmeinungen und neuen Entwicklungen. Um diesen Vorteil optimal zu nutzen, ist es essenziell, berufliche und private Accounts zu trennen. So kann man den Algorithmus gezielt auf wissenschaftliche Inhalte trainieren und fokussieren, ohne Ablenkung durch persönliche Interessen. Dies hilft, die eigene Aufmerksamkeit effizient auf fachlich relevante Themen zu lenken.

## Vernetzung und Karriereentwicklung

Soziale Medien sind heute zentrale Instrumente für Vernetzung und Karriereentwicklung in der Radiologie. Sie ermöglichen den weltweiten Austausch mit Fachkolleg:innen, fördern Kooperationen und wissenschaftliche Diskussionen. Besonders LinkedIn hat in den letzten Jahren an Bedeutung gewonnen – die Kongressbeiträge am Beispiel ECR haben sich dort laut eigener Recherche zwischen 2023 und 2024 fast verdreifacht. Eine detaillierte Analyse des Nutzer:innenverhaltens in der Radiologie steht jedoch noch aus. Besonders für Nachwuchsradiolog:innen erleichtern diese Netzwerke den Zugang zu Mentoring-Programmen und Stipendien, sowie die Interaktion mit etablierten nationalen und internationalen Radiolog:innen.

## Wissenschaftskommunikation

Studien zeigen, dass die aktive Nutzung sozialer Medien die Zitierhäufigkeit wissenschaftlicher Publikationen erhöhen kann [2]. Dies hat nahezu alle führenden radiologischen Fachzeitschriften veranlasst, Social Media gezielt für die Wissenschaftskommunikation einzusetzen. So wurden spezielle Redaktionsposten für Social-Media-Beauftragte geschaffen, und viele Artikel erhalten multimediale Zusammenfassungen (Podcasts, Videos, Bilder) zur besseren Sichtbarkeit [3]. Durch präzise und verständliche Kommunikation lassen sich komplexe Inhalte effektiv an das eigene Netzwerk vermitteln. Bestimmte Hashtags oder Markierungen ermöglichen zudem eine Reichweite über das eigene Netzwerk hinaus. Ein interessanter Aspekt: Kongresspräsenz und Posting-Aktivität in sozialen Medien scheinen miteinander zu korrelieren [4].

## Weiterbildung

Neben der Netzwerkpflege und der Verbreitung eigener Forschungsergebnisse spielt für viele Nutzer:innen sozialer Medien die persönliche Weiterbildung eine zentrale Rolle [5].

Im Vergleich zu *klassischen* digitalen Lernanwendungen bieten soziale Medien interaktiven Austausch, Echtzeit-Updates zu Fachthemen und individuell anpassbare Lernressourcen.

Allerdings gibt es auch Herausforderungen: Die wissenschaftliche Qualität der Inhalte ist nicht immer gesichert, Fehlinformationen können sich schnell verbreiten, und die Fülle an Inhalten kann eine gezielte Auswahl relevanter Informationen erschweren. Schließlich besteht die Gefahr, dass Algorithmen nur einseitige oder populäre Inhalte bevorzugen (was für die Nutzer:in nur bedingt nachvollziehbar ist, je nach Plattform), wodurch eine kritische Auseinandersetzung mit unterschiedlichen Perspektiven erschwert (wenn nicht gar unmöglich) wird.

## Potenzielle Fallstricke und Folgen

Die Nutzung von Social Media birgt jedoch auch Risiken [3]: Provokative Inhalte oder kontroverse Themen können zwar die eigene Reichweite steigern, aber auch das eigene Image schädigen und berufliche Chancen beeinträchtigen. Beim Teilen fremder Inhalte ist eine sorgfältige Prüfung der Richtigkeit sowie die korrekte Quellenangabe essenziell, um „Fake News“ zu vermeiden. Radiolog:innen sollten bedenken, dass Social-Media-Posts dauerhaft sind, von potenziellen Arbeitgebern eingesehen und von Kolleg:innen unterschiedlich bewertet werden können. Besonders beim Selbstmarketing – etwa durch „Humble-Bragging“ (verstecktes Eigenlob) – ist Fingerspitzengefühl gefragt, da die Wahrnehmung kulturell variieren kann. Eine zweite Meinung vor dem Posten kann helfen, Fehltritte zu vermeiden.

Zudem werden zunehmend medizinische Fallbeispiele auf Social Media geteilt. Dabei sind Datenschutz und Vertraulichkeit essenziell: Patientendaten müssen anonymisiert werden, und eine Veröffentlichung sollte nur mit ausdrücklicher Zustimmung der Patient:innen erfolgen, um ethische und rechtliche Probleme zu vermeiden.

## Fazit für die Praxis

- Soziale Medien bieten Radiolog:innen vielfältige Möglichkeiten zur Vernetzung, Karriereentwicklung und wissenschaftlichen Sichtbarkeit.
- Sie bieten zudem schnellen Zugang zu Fachinformationen und interaktiven Lernressourcen, erfordern aber kritische Bewertung.
- Dauerhafte Sichtbarkeit von Beiträgen kann berufliche Chancen beeinflussen.
- Korrekte Quellenangaben, sensibler Umgang mit Inhalten und bewusste Selbstpräsentation sind deshalb entscheidend.
